# Rapamycin protects aging muscle

**DOI:** 10.18632/aging.102176

**Published:** 2019-08-27

**Authors:** Huibin Tang, Joseph B. Shrager, Daniel Goldman

**Affiliations:** 1 Department of Cardiothoracic Surgery, Stanford University School of Medicine, Stanford, CA 94305, USA; 2 VA Palo Alto Healthcare System, Palo Alto, CA 94304, USA; 3 Molecular and Behavioral Neuroscience Institute, Department of Biological Chemistry, University of Michigan, Ann Arbor, MI 48109, USA

**Keywords:** rapamycin, mTORC1, aging, skeletal muscle, oxidative stress

As life expectancy increases, age-related health issues will continue to rise. One of the central health issues among the elderly is age-related progressive muscle loss and functional decline, i.e., sarcopenia. Sarcopenia causes frailty and falls, and thereby it may bring about a series of complications that increases morbidity and mortality. Successful interventions against sarcopenia may help improve physical strength and quality of life for the elderly population.

A decade ago, rapamycin, an mTORC1 inhibitor, was reported to extend the lifespan of mice [1]. Consistent with this, rapamycin and other mTORC1 inhibitors also protect several organs/tissues against age-related functional decline [2]. Rapamycin’s effect on aging skeletal muscle, however, was not explored until recently. It has long been known that mTORC1 activity is induced in aging muscle. Two recent reports with genetic and pharmacological evidence reveal two important findings: *1)* *Chronic activation of mTORC1 stimulates progressive muscle damage and loss*. Interestingly, when TSC1 knockout mice were used to activate mTORC1, enlarged muscle fibers were noted starting at approximately 2 months of age. However, this seemingly beneficial effect ultimately morphed into muscle fiber damage with mixed hypertrophic and atrophic fibers seen at ~ 6 - 9 months of age, and it ultimately resulted in ~ 50% loss of muscle mass at 18 months of age. This significant muscle loss is accompanied by a reduced survival rate of TSC1 knockout mice, which exhibit a median lifespan of 18 months [3]. *2) Inhibition of mTORC1 with rapamycin prevents age-related muscle loss*. Consistent with the observation that the hyperactive mTORC1 induces muscle damage and loss, inhibition of mTORC1 activity with rapamycin or rapalogs protects aging muscle from atrophy in mice and rats. For example, treatment with rapamycin at 14ppm, from 9 months to 30 months of age, reduced apoptosis and promoted retention of peripherally located nuclei, and this was associated with reduced fiber loss in aging skeletal muscle [3]. In a separate study, a shorter duration of rapalog (RAD001) treatment at the dose of 0.15mg/kg for 6-weeks, starting from 22 month of age, preserved both fiber size and muscle weight [4]. These data suggest that mTORC1 is necessary and sufficient to drive skeletal muscle aging.

**Figure 1:**
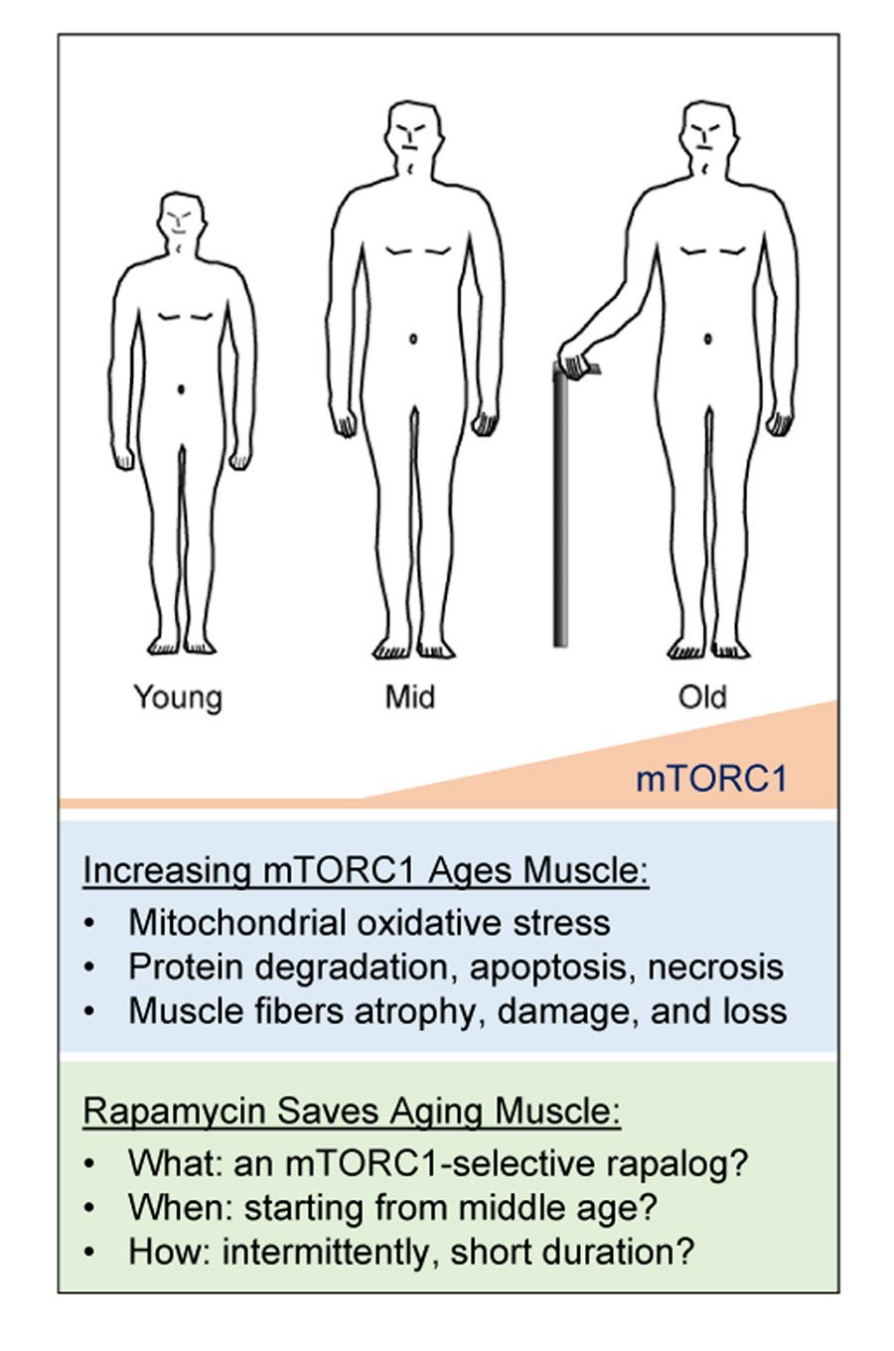
**A hypothetical view of the etiology and therapy of sarcopenia.** mTORC1 activity increases during aging, starting from middle age, resulting in progressively altered mitochondria, leading to mitochondrial oxidative stress and thus catabolism including protein degradation, apoptosis, and necrosis. This elevated catabolic activity results in muscle fiber loss, atrophy, and damage. Therefore, inhibition of mTORC1 activity with rapamycin may rescue skeletal muscle during aging. mTORC1-selective inhibitors and optimized treatment protocols may maximize the beneficial effect of rapalog treatment.

Do these findings conflict with the current understanding of the anabolic function of mTORC1? We do not think so. We believe the ultimate effect of mTORC1 depends upon: 1) how mTORC1 is activated, and 2) for how long mTORC1 remains activated. Akt-dependent, short-term activation of mTORC1 appears usually to be beneficial to cells, whereas Akt-independent, chronic activation of mTORC1 appears to be detrimental to cells. It is well known that mTORC1 is an important anabolic regulator that is essential for cell growth, regeneration, and maintenance of muscle mass [5,6]. Akt-dependent activation of mTORC1 (e.g., through increasing IGF or Akt activity), prevents muscle atrophy [7]. In contrast to this prevailing knowledge, Akt-independent, chronic activation of mTORC1 (e.g., muscle-specific knockout of TSC1) appears to trigger protein degradation and muscle atrophy, through a cascade of downstream events that includes: 1) feedback inhibition of Akt and the consequent activation of FoxO1; 2) induction of GDFs; 3) inhibition of autophagy; and 4) increased mitochondrial oxidative stress [3,8]. The most striking ultrastructural change is the presence of morphologically and functionally abnormal mitochondria in muscle fibers with chronic activation of mTORC1. These gigantic, but dysfunctional mitochondria probably resulted from mTORC1-driven mitochondrial proliferation and mTORC1-mediated inhibition of mitophagy. Over time, the accumulation of these abnormal mitochondria may lead to the induction of mitochondrial oxidative stress, which, we believe, is the pivotal mediator of mTORC1-induced catabolism that leads to protein degradation and apoptotic/necrotic cell death. Thus, Akt-independent activation of mTORC1 induces catabolism in addition to its conventional anabolic activity. The progressive myopathy seen in the mTORC1-hyperactive muscle is the net outcome of a complex process that perturbs the balance between anabolism and catabolism, with catabolism winning out and leading to muscle fiber damage and loss. This hypothesis explains the coexistence of hypertrophic (anabolism > catabolism) and atrophic (catabolism > anabolism) fibers, as well as the progressive nature of mTORC1-induced muscle damage. It also implies that an early preventive treatment (perhaps starting in middle age when mTORC1 activity increases, at least in mice) with mTORC1 inhibitors may help prevent sarcopenia (Figure 1). This hypothesis is also in some ways consistent with the “mitochondrial theory of aging” (Harman D. 1972), but our mTORC1-centric view advances that theory by identifying mTORC1 as a specific inducer of mitochondrial oxidative stress in aging skeletal muscle.

Additional work building on these studies needs to be done which would include: 1) Developing selective mTORC1 inhibitors. Selective mTORC1 inhibitors may minimize side effects that may result from rapamycin-induced suppression of mTORC2, a known activator of Akt. 2) Developing optimal protocols for rapamycin treatment. Optimized dose, dosing frequency, and dosing intervals may help achieve maximum beneficial effect. As shown in recent reports, long-term (~21 month) rapamycin treatment at 14ppm failed to prevent age-related decline of muscle fiber size, whereas a short-term (6 week) rapamycin treatment at 0.15mg/kg succeeded. 3) Investigating rapamycin-dependent changes in muscle contractile function, and the regenerative capacity of satellite cells. 4) Identification and characterization of the function of upstream regulators and the downstream effectors of mTORC1. In addition, although we have already identified the transcriptional targets, GDFs (esp. GDF15), and the posttranslational target, FoxO1, downstream of mTORC1’s activation, their functional roles in aging skeletal muscle remains to be elucidated. 5) Developing a better understanding of the relationship between mTORC1/rapamcyin and other age-related signaling pathways (e.g., sirtuins/NAD+) in skeletal muscle.

This mTORC1-centric view of skeletal muscle aging helps to unify many of the previously known actors in aging -- aberrant mitochondria, free radicals/oxidative stress, autophagy, protein degradation, FoxO, and calorie restriction. Thus, mTORC1 potentially represents a key, central driver of aging. Since the components in the mTORC1 signaling cascade are ubiquitously expressed, the same mechanistic framework – i.e., the mTORC1-mitochondria oxidative stress-catabolism axis --may underlie the aging process more generally. Rapamycin, as a well-known mTORC1 inhibitor, appears to positively impact aging skeletal muscle in mice and rats. We may be approaching a time to move to clinical studies of rapamycin’s effect on aging human skeletal muscle.
